# Gastric Emphysema after Intubation

**DOI:** 10.5334/jbr-btr.812

**Published:** 2017-01-10

**Authors:** Valeria Angelino, Giovanni Volpicelli, Luciano Cardinale

**Affiliations:** 1 Department of Radiology, San Luigi Gonzaga Hospital, Orbassano (Torino), Italy; 2 Department of Emergency Medicine, San Luigi Gonzaga Hospital, Orbassano (Torino), Italy

**Keywords:** Gastric emphysema, nasogastric tube, emphysematous gastritis

A 72-year-old male with history of psychiatric disorders was referred to our emergency department in a state of cardiac arrest after a suicide attempt. The patient had attempted suicide by a knife of 30 cm in length. He reported a deep wound to the left side of the neck, together with superficial abdominal wounds. During transportation to the hospital, the patient was orally intubated and cardio-pulmonary resuscitation and fluids were administered with restoration of hemodynamic profile. After arrival, he was stabilized by fluid treatment and blood transfusions.

Once stabilized and while ventilated, decision was taken to perform thoracic-abdominal multiphasic contrast-enhanced CT examination to assess further traumatic damages. At the preliminary digital tomogram, a thin, linear area of increased radiolucency that outlines the gastric wall was imaged in the upper left abdominal region (Figure 1, arrow). At CT scan, gastric intramural gas was clearly detected (Figures 2, 3, coronal reformatted and axial images, arrows); furthermore a small amount of retroperitoneal free air was seen around splenic vein and diaphragmatic crura (Figure 3). The diagnosis of gastric emphysema due to mild mucosal disruption was concluded.

**Figure 1:**
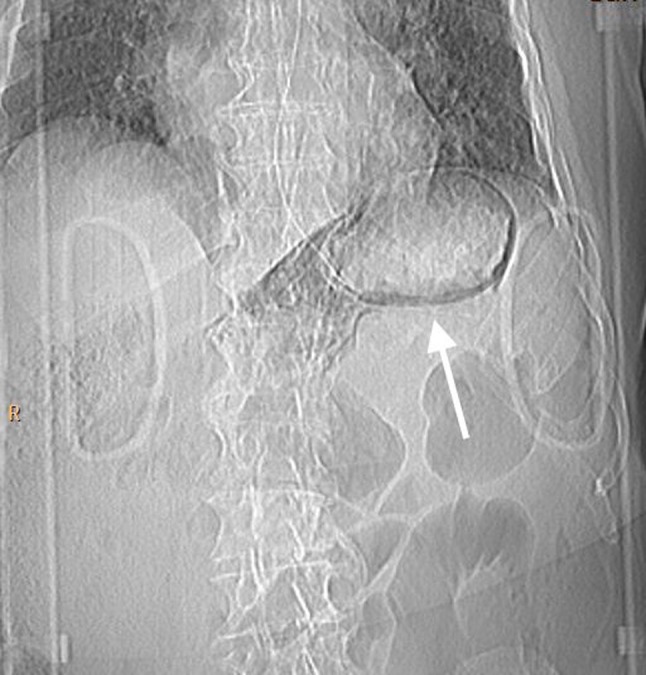
Topogram preliminary to the CT scan. Linear gas shadows in the upper left abdominal region (arrow), apparently round the stomach.

**Figure 2:**
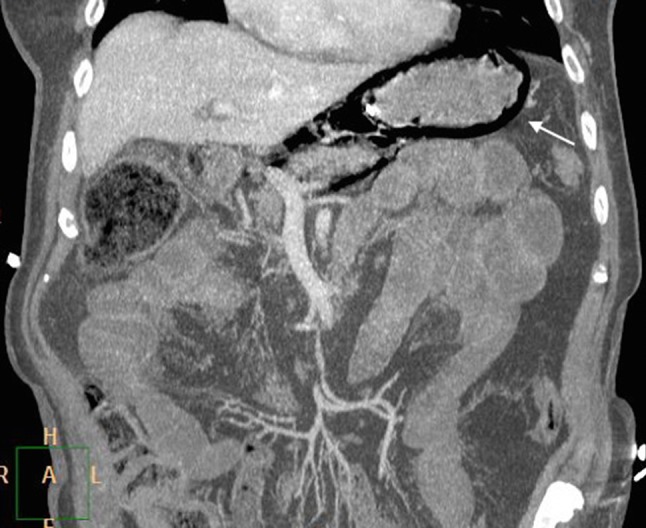
Contrast-enhanced CT, MPR reformats. Large amounts of intramural gas in the stomach (arrow) with no evidence of free peritoneal air.

**Figure 3:**
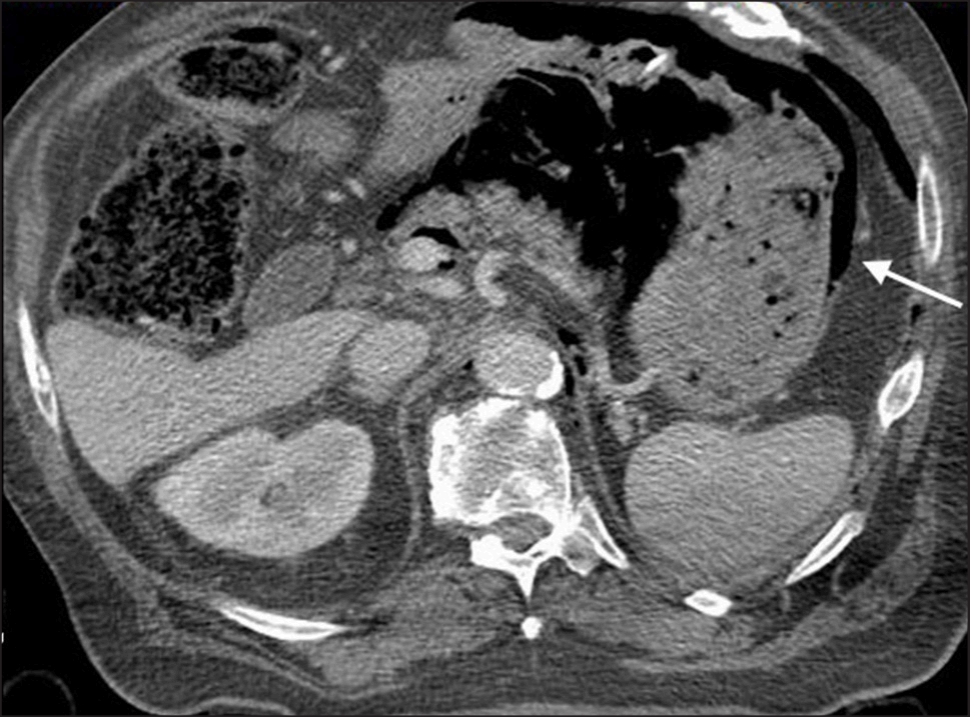
Contrast-enhanced CT, axial slice. Evidence of gastric intramural gas (arrow) and air bubbles within the retroperitoneal space.

The most likely explanation of the mucosal trauma was an excess of pressure inside the stomach caused by the attempts of intubation in the ambulance and some air insufflation in the esophageal lumen. The patient was managed conservatively and his condition slowly recovered during the following days.

## Comment

Three conditions of gas collection in the stomach wall have been described: interstitial gastric emphysema, cystic pneumatosis, and emphysematous gastritis [1].

Interstitial gastric emphysema is seen when air from an extrinsic source enters the stomach wall and accumulates in the submucosa, subserosa, or both. The development of intramural air is frequently preceded by gastric distension and vomiting. The main causes include gastric obstruction due to pyloric stenosis, carcinoma of the antrum, gastric volvulus, and partial or complete duodenal obstruction secondary to pancreatic or ampullary carcinoma, duodenal stenosis, gall stones. Other main causes are penetrating gastric ulcers, nasogastric tube placement, endoscopy with air insufflation and rupture of pulmonary bullae or pneumothorax [2]. Radiographs characteristically demonstrate a linear area of increased radiolucency conforming to the gastric contours.

Cystic pneumatosis refers to the radiographic observation of multiple 1- to 2-mm gas-filled cysts in the wall of the stomach and the bowel of patients with mild gastrointestinal symptoms.

Emphysematous gastritis occurs when there is diffuse infiltration of the stomach wall by pathogenic gas-forming bacteria and it is often associated with parietal thickening and other findings secondary to sepsis (portal axis thrombosis/air infarction of different abdominal viscera).
